# Longitudinal associations between multiple health-risk indicators and psychological distress among university students: a three-wave cross-lagged study

**DOI:** 10.3389/fpubh.2026.1958845

**Published:** 2026-09-23

**Authors:** Yulong Sun, Meng Qin, Yang Qin, Qifu Wang

**Affiliations:** 1 School of Physical Education and Sports, Guangxi University, Nanning, China; 2 School of Physical Education, Guangzhou Sport University, Guangzhou, China; 3 Department of Physical Education, Guilin University of Electronic Technology, Guilin, China; 4 School of Sports Science, Changsha Normal University, Changsha, China

**Keywords:** cross-lagged panel model, latent growth model, longitudinal study, multiple health-risk indicators, psychological distress, university students

## Abstract

**Background:**

Multiple health-risk indicators (MHRIs) and psychological distress may accumulate together among university students, yet longitudinal evidence regarding their developmental trajectories and bidirectional associations remains limited. This study examined changes in MHRIs and psychological distress and their bidirectional longitudinal associations to provide evidence for integrated risk identification and intervention strategies among university students.

**Methods:**

A three-wave longitudinal study was conducted among university students from nine universities in Guangxi, Guizhou, and Yunnan provinces in April 2025, October 2025, and April 2026. The final analytic sample comprised 1,762 students who completed all three waves and had consistent anonymous codes. MHRIs count was calculated by summing nine behavioral, psychosocial, and health-related risk indicators. Psychological distress was assessed using the Depression Anxiety Stress Scales-21 (DASS-21). Latent growth models (LGMs) were used to examine developmental trajectories and individual differences in initial levels and rates of change, and a cross-lagged panel model (CLPM) was applied to assess bidirectional longitudinal associations after adjusting for baseline covariates, including sex, household registration, campus residence status, only-child status, and adverse childhood experiences.

**Results:**

MHRIs count and psychological distress both showed increasing trends across the three measurement occasions. Unconditional LGMs indicated significant increases over time with individual differences in initial levels and rates of change, corresponding to average increases of 0.10 MHRIs and 1.55 psychological distress points per 6-month interval. The CLPM demonstrated acceptable-to-good model fit. After adjustment for baseline covariates, higher psychological distress predicted a greater number of subsequent MHRIs at both follow-up intervals (T1–T2: β = 0.167, *p* < 0.001; T2–T3: β = 0.141, *p* < 0.001). Conversely, higher MHRIs also predicted subsequent increases in psychological distress (T1–T2: β = 0.132, *p* < 0.001; T2–T3: β = 0.119, *p* < 0.001).

**Conclusion:**

MHRIs and psychological distress increased over the 1-year follow-up period and demonstrated a stable positive bidirectional longitudinal association. Integrating psychological distress screening with assessment of multiple health risks, continuous monitoring, and coordinated health promotion strategies may help reduce the accumulation of psychological and health-related risks among university students.

## Introduction

The university period is a critical stage during which lifestyles, health-related behaviors, and psychosocial characteristics are developed and consolidated (1). Previous studies have shown that multiple health risks are common among university students, with a recent large-scale study among Chinese college students reporting that more than 60% of students experienced two or more health-risk behaviors (2). Different types of behavioral risks, psychosocial risks, and health-related risks often do not occur independently but tend to accumulate and co-occur at the individual level (3, 4). With increasing autonomy and growing academic, interpersonal, and employment-related pressures, multiple health risks, including substance use, sleep problems, problematic internet use, and psychosocial difficulties, may further cluster among university students (2–4). The coexistence of multiple health risks not only reflects a greater overall health burden but is also associated with impaired physical and psychological functioning, poor academic adjustment, and adverse long-term health outcomes (5, 6). Therefore, investigating multiple health-risk indicators (MHRIs) and their developmental changes among university students has important public health implications for risk identification and integrated intervention in university settings.

MHRIs refer to the simultaneous presence of multiple behavioral risks, psychosocial risk exposures, and health-related risks that may adversely affect health (7). Compared with examining individual risk factors separately, an indicator accumulation approach provides a more comprehensive assessment of overall health risk burden and better reflects the co-occurrence of different types of risks in real-world contexts (8, 9). During university years, changes in living environments, self-management abilities, and social interaction patterns may contribute to changes in the level of multiple health risks over time. However, comparisons of average levels across different time points cannot fully capture developmental processes, and further investigation is needed to examine overall trajectories and individual differences in initial levels and rates of change.

Psychological distress may represent an important psychological factor for understanding MHRIs among university students (10). MHRIs may be associated with subsequent psychological distress through pathways involving disrupted sleep patterns, impaired academic functioning, interpersonal difficulties, and reduced social support. Conversely, persistent depressive, anxiety, and stress symptoms may undermine self-regulation capacity and health-related decision-making, thereby increasing the likelihood of unhealthy behaviors and other health-related risks (11–13). Therefore, MHRIs and psychological distress may exhibit dynamic and mutually influential associations, highlighting the need for longitudinal research to examine their developmental changes and bidirectional associations.

Previous studies have indicated that health risks among university students do not occur in isolation but represent the combined influence of multiple behavioral risks, psychosocial risks, and health-related risks. Existing evidence suggests that health-related risks, including smoking, alcohol use, unhealthy dietary behaviors, and sleep problems, often cluster together and are closely associated with depression, anxiety, and psychological distress (14, 15). Furthermore, longitudinal research has suggested that the accumulation of unhealthy lifestyle factors may have persistent associations with mental health problems and may involve bidirectional influences over time (16). However, several gaps remain in the existing literature. First, previous longitudinal studies have mainly focused on individual health-risk behaviors or specific risk combinations, with limited attention to the overall accumulation and dynamic changes of multiple health-risk indicators encompassing behavioral, psychosocial, and health-related domains. Second, although some studies have examined changes in health-risk behaviors over time, they have primarily focused on differences in average levels across measurement occasions and rarely investigated individual differences in initial risk levels and rates of change. Thus, whether MHRIs follow distinct developmental trajectories during university years remains insufficiently understood. Finally, although previous studies have identified close associations between psychological distress and multiple health risks, the temporal ordering and bidirectional longitudinal associations between them require further investigation.

Therefore, addressing the limitations in comprehensive health-risk assessment, developmental patterns, and the dynamic relationship between psychological distress and health risks, this study employed a three-wave longitudinal design to examine the developmental trajectories of MHRIs and psychological distress and their longitudinal associations among university students. Unlike previous studies that primarily focused on individual health-risk behaviors, this study constructed a composite MHRIs measure from the perspective of overall risk burden to capture the co-development of multiple behavioral, psychosocial, and health-related risks. Furthermore, latent growth models were applied to examine developmental changes and individual differences in these indicators, and cross-lagged panel models were used to investigate the bidirectional longitudinal associations between MHRIs and psychological distress. This study provides new longitudinal evidence to support the dynamic identification of health risks and the integration of psychological and behavioral risk interventions among university students.

## Materials and methods

### Participants and study design

This three-wave longitudinal study used cluster random sampling to select universities and classes from general higher education institutions in Guangxi, Guizhou, and Yunnan provinces. Three universities were randomly selected in each province. Before sampling, a list of eligible general higher education institutions in each province was obtained. Universities were assigned sequential numbers, and a computer-generated randomization procedure was used to select three universities from each province. At each university, two classes were randomly selected from humanities disciplines and two from science disciplines, resulting in 36 classes.

The required sample size was estimated before data collection based on structural equation modeling requirements for longitudinal studies. Considering the complexity of the latent growth models and cross-lagged panel models, a small-to-moderate effect size (approximately standardized effect size of 0.10–0.30), a two-sided significance level of 0.05, and a statistical power of 0.80 were assumed. The minimum required sample size was estimated to be approximately 1,200 participants. Considering the design effect associated with cluster sampling and potential attrition across the three waves, the target baseline sample size was increased to approximately 1,500 participants.

The inclusion criteria were as follows: (1) enrollment as a full-time student at a general higher education institution; (2) ability to understand the questionnaire and complete it independently; (3) voluntary participation and provision of informed consent; and (4) ability to generate an anonymous code according to a standardized rule for matching data across the three waves. The exclusion criteria were as follows: (1) severe cognitive impairment or mental illness that prevented independent questionnaire completion; (2) missing key information during the survey; (3) evident patterned responding, logical inconsistencies, or an abnormal completion time; and (4) anonymous codes that could not be matched across the three waves.

The three surveys were conducted in April 2025, October 2025, and April 2026. A total of 1,922 university students were enrolled at baseline. At each wave, questionnaires were distributed to the participants identified at baseline. Valid questionnaires were returned by 1,841, 1,828, and 1,794 participants at T1, T2, and T3, respectively. After excluding records with missing data, invalid questionnaires, and anonymous codes that could not be matched, the final analytic sample comprised 1,762 students who completed all three waves and had consistent questionnaire codes. The complete follow-up retention rate was 91.68%.

### Survey procedures and quality control

A standardized survey procedure was used. Before data collection, the research team provided uniform training to site coordinators and survey administrators. The training covered the study objectives, questionnaire completion requirements, informed-consent procedures, anonymous coding rules, data-confidentiality requirements, and procedures for responding to questions on site. Before each survey, administrators explained the study objectives and the principles of anonymity, confidentiality, and voluntary participation. All participants completed the questionnaire independently after providing informed consent. To protect privacy while enabling longitudinal matching, the study did not collect direct identifiers such as names, student identification numbers, or national identification numbers. Instead, students generated anonymous codes according to a standardized rule; these codes were used solely to match data across T1, T2, and T3.

Quality control was implemented during both survey administration and data processing. During administration, standardized instructions, mandatory-response settings, and logic checks were used to reduce missing and invalid responses. During data processing, questionnaire completeness, completion time, variable ranges, patterned responding, duplicate submissions, and consistency of anonymous codes were checked. Records with missing core variables, evident logical inconsistencies, abnormal responding, or codes that could not be matched across the three waves were excluded, yielding a complete, matched three-wave dataset.

### Measures and variable definitions

The analysis included demographic variables, multiple health-risk indicator (MHRIs) count, and psychological distress. Demographic and background variables were included as baseline covariates in the CLPM, whereas MHRIs count and psychological distress were measured repeatedly at T1, T2, and T3. Because assessments were conducted every 6 months, the recall periods of selected instruments were standardized to the past 30 days, following previous longitudinal studies, to reduce recall bias and overlap between measurement windows associated with longer recall periods (17). Variable coding is presented in Table 1.

**Table 1:** Coding and ranges of study variables.

Variable	Type	Coding or range
Multiple health-risk indicator count	Count	0–9;number of co-occurring multiple health-risk indicators present
Psychological distress score	Continuous	0–126; higher scores indicate greater psychological distress
Depression	Continuous	0–42
Anxiety	Continuous	0–42
Stress	Continuous	0–42
Age	Continuous	Age in completed years
Sex	Categorical	0 = male; 1 = female
Household registration	Categorical	0 = urban; 1 = rural
Campus residence status	Categorical	0 = on campus; 1 = off campus
Only-child status	Categorical	0 = no; 1 = yes
Adverse childhood experiences	Categorical	0 = absent; 1 = present

### Demographic characteristics

A self-developed general information questionnaire was used to collect demographic and background information, including age, sex, household registration, campus residence status, only-child status, and adverse childhood experiences.

Adverse childhood experiences (ACEs) were assessed using the Adverse Childhood Experiences Questionnaire (18). The 10 items cover emotional abuse, physical abuse, sexual abuse, emotional neglect, physical neglect, and household dysfunction. Each item was coded 0 if the event had not been experienced and 1 if it had. Total scores ranged from 0 to 10, and an ACE total score ≥ 1 indicated the presence of any ACE.

#### Multiple health-risk indicators

The health-risk indicators were defined primarily according to commonly used criteria in surveys of health-related risks among adolescents and university students and were operationalized in accordance with the study questionnaire. Nine behavioral, psychosocial, and health-related risk indicators were included: smoking, alcohol use, frequent picky eating, insomnia symptoms, traditional bullying victimization, cyberbullying victimization, nonsuicidal self-injury (NSSI), Internet gaming disorder (IGD), and problematic social media use (PSMU). Each indicator was dichotomized, with absence coded as 0 and presence or a positive screen coded as 1.

Smoking, alcohol use, and picky eating were each assessed with a single item (19). Smoking on at least 1 day during the past 30 days was classified as smoking, and alcohol consumption on at least 1 day during the past 30 days was classified as alcohol use. Picky eating was assessed on a 5-point scale (0 = never, 1 = occasionally, 2 = sometimes, 3 = often, and 4 = always), with higher scores indicating more frequent picky eating. Responses of “often” or “always” were classified as frequent picky eating.

Insomnia symptoms were assessed using the Insomnia Severity Index (ISI) (20). The seven items assess difficulty initiating sleep, difficulty maintaining sleep, early-morning awakening, satisfaction with sleep, interference of sleep problems with daytime functioning, noticeability of sleep problems to others, and distress caused by sleep problems during the past 30 days. Items are rated on a 5-point scale, yielding a total score of 0–28; higher scores indicate more severe insomnia symptoms. Following previous research, participants with an ISI total score ≥ 8 were classified as screening positive for insomnia symptoms. Cronbach's alpha values at the three waves were 0.91, 0.92, and 0.91, respectively.

Traditional bullying victimization was assessed using the traditional bullying victimization items from the Delaware Bullying Victimization Scale–Student (21). The 12 items assess experiences of physical, verbal, and relational bullying victimization during the past 30 days. Items are rated on a 6-point scale, yielding a total score of 0–60; higher scores indicate more frequent traditional bullying victimization. Following previous research, a score ≥ 1 on any item indicated traditional bullying victimization. Cronbach's alpha values at the three waves were 0.92, 0.88, and 0.90, respectively.

Cyberbullying victimization was assessed using the cyberbullying victimization items from the Delaware Bullying Victimization Scale–Student (22). The six items assess experiences of online insults, threats, exclusion, and malicious dissemination of information during the past 30 days. Items are rated on a 6-point scale, yielding a total score of 0–30; higher scores indicate more frequent cyberbullying victimization. Following previous research, a score ≥ 1 on any item indicated cyberbullying victimization. Cronbach's alpha values at the three waves were 0.87, 0.85, and 0.86, respectively.

NSSI was assessed using the self-injury behavior items from the Chinese version of the Functional Assessment of Self-Mutilation (C-FASM) (23). The 10 items assess self-injurious behaviors without clear suicidal intent during the past 30 days, including cutting, biting, burning, pinching, scratching, hitting the body, and pulling hair. Items are rated on a 5-point scale, yielding a total score of 0–40; higher scores indicate more frequent self-injurious behavior. Following previous research, a C-FASM total score ≥ 1 indicated NSSI. Cronbach's alpha values at the three waves were 0.82, 0.87, and 0.85, respectively.

IGD was assessed using the Internet Gaming Disorder Scale–Short Form (IGDS9-SF) (24). The nine items assess gaming-related loss of control, tolerance, withdrawal, persistent use, functional impairment, and conflict during the past 30 days. Items are rated on a 5-point scale, yielding a total score of 9–45; higher scores indicate greater risk of IGD. Following previous research, participants with an IGDS9-SF total score ≥ 32 were classified as screening positive for probable IGD. Cronbach's alpha values at the three waves were 0.94, 0.91, and 0.94, respectively.

PSMU was assessed using the Bergen Social Media Addiction Scale (BSMAS) (25). The six items assess the core addiction features of salience, mood modification, tolerance, withdrawal, conflict, and relapse during the past 30 days. Items are rated on a 5-point scale, yielding a total score of 6–30; higher scores indicate more problematic social media use. Based on relevant literature and the variable definition used in this study, participants with a BSMAS total score ≥ 24 were classified as screening positive for PSMU (26). Cronbach's alpha values at the three waves were 0.89, 0.92, and 0.88, respectively.

A multiple health-risk indicator count (MHRIs count) was calculated from the nine binary indicators described above. The indicators were summed to yield a score ranging from 0 to 9, with higher scores indicating a greater number of co-occurring multiple health-risk indicators.

#### Psychological distress

Psychological distress was assessed using the Depression Anxiety Stress Scales-21 (DASS-21) (27). The 21 items comprise three dimensions: depression, anxiety, and stress symptoms. Items are rated on a 4-point scale. The DASS-21 total score was calculated by summing the item scores and multiplying the sum by 2, yielding a range of 0–126; higher scores indicate greater psychological distress.

DASS-21 total scores were calculated at T1, T2, and T3 and used as the primary measure of psychological distress in the latent growth and cross-lagged panel models. Previous studies have demonstrated good reliability and validity for the scale. Cronbach's alpha values at the three waves were 0.91, 0.93, and 0.93, respectively.

### Statistical analysis

Statistical analyses were conducted using IBM SPSS Statistics 29.0 and Mplus 8.3. First, the primary questionnaire items at T1, T2, and T3 were entered separately into unrotated principal component analyses, and Harman's single-factor test was used to assess common method bias. A first factor accounting for less than 40% of the variance was taken to indicate that common method bias was not substantial. Descriptive analyses were then conducted for participant demographic characteristics, DASS-21 total scores, MHRIs count, and the prevalence of each health-risk indicator. Categorical variables are presented as frequencies and percentages, and continuous variables as means and standard deviations. Pearson correlation analyses examined associations between DASS-21 total scores and MHRIs count across the three waves.

Before longitudinal modeling, longitudinal measurement invariance was evaluated at the DASS-21 item level. Because the DASS-21 items are ordinal categorical variables, models were estimated using the weighted least squares mean and variance adjusted estimator (WLSMV). Configural, metric, and threshold invariance were tested sequentially. The configural model specified the same factor structure across the three waves; the metric model additionally constrained the factor loadings of corresponding items to equality; and the threshold model further constrained the thresholds of corresponding items to equality. Changes of ΔCFI <0.010 and ΔRMSEA <0.015 between adjacent models were considered to support longitudinal measurement invariance (28). The measurement-invariance results are reported in the Supplementary Material.

Unconditional latent growth models (LGMs) were constructed using MHRIs count and DASS-21 total scores at T1, T2, and T3 as observed variables. The LGMs were used to characterize the developmental trajectories of MHRIs count and psychological distress and to examine individual differences in initial levels and rates of change. The linear slope factor loadings were fixed at 0, 1, and 2, representing baseline, 6-month follow-up, and 12-month follow-up, respectively. The intercept factor represented the initial level, whereas the slope factor represented the average rate of change per 6-month interval. The means of the intercept and slope factors were used to describe the overall initial levels and developmental trends; their variances were used to evaluate individual differences in initial levels and rates of change; and the correlation between the intercept and slope factors was estimated. MHRIs count was a summed count variable derived from nine indicators, ranging from 0 to 9. Because this study focused on longitudinal changes in the overall burden of multiple health risks and their temporal associations, MHRIs count was treated as an approximately continuous observed variable in the LGM and CLPM analyses, consistent with previous research (29, 30).

Subsequently, a cross-lagged panel model (CLPM) was constructed to examine the temporal ordering and bidirectional longitudinal associations between MHRIs count and psychological distress across adjacent time points. The model included autoregressive paths between adjacent waves, bidirectional cross-lagged paths, and within-wave residual correlations between MHRIs count and psychological distress. The autoregressive and cross-lagged paths were freely estimated across both time intervals. Baseline MHRIs count and psychological distress were regressed on sex, household registration, campus residence status, only-child status, and adverse childhood experiences to control for baseline demographic and background differences. The latent growth models (LGMs) and cross-lagged panel model (CLPM) were estimated using robust maximum likelihood estimation (MLR) to reduce the influence of non-normality on parameter estimates and standard errors (31). Given the clustered sampling design, intraclass correlation coefficients (ICCs) were estimated at the university and class levels to evaluate the extent of clustering.

Model fit was evaluated using χ^2^, the comparative fit index (CFI), the Tucker–Lewis index (TLI), the root mean square error of approximation (RMSEA), and the standardized root mean square residual (SRMR). CFI and TLI values ≥ 0.90 and RMSEA and SRMR values ≤ 0.08 were considered to indicate acceptable fit (32). Standardized parameter estimates, standard errors, and statistical significance are reported. All statistical tests were two-sided, with α = 0.05.

### Ethics statement

This study was conducted as part of an ethically approved longitudinal project on psychological and behavioral health among university students. Ethical approval was obtained from the Human Research Ethics Committee of Changsha Normal University (approval no. 2025-0302).

## Results

### Common method bias and attrition analyses

Harman's single-factor test was used to assess common method bias. Unrotated principal component analyses extracted seven, seven, and six factors with eigenvalues greater than 1 at T1, T2, and T3, respectively. The first unrotated factor accounted for 25.26, 29.46, and 30.52% of the variance at T1, T2, and T3, respectively, all below the 40% criterion, suggesting no substantial common method bias.

Baseline characteristics were compared between participants who completed all three waves and those who did not. No statistically significant baseline differences were observed in sex, household registration, campus residence status, only-child status, adverse childhood experiences, baseline MHRIs count, or baseline psychological distress (all *p* > 0.05), providing no evidence of substantial selective attrition. The final analysis included 1,762 university students with complete data and consistent codes across all three waves.

### Participant characteristics

The study included 1,762 university students who completed all three waves and had consistent anonymous codes. Their mean age was 19.03 years (SD = 0.84). Of the participants, 957 (54.31%) were female, 1,169 (66.35%) had rural household registration, 166 (9.42%) lived off campus, 512 (29.06%) were only children, and 298 (16.91%) reported at least one adverse childhood experience.

At baseline, the mean MHRIs count was 1.41 (SD = 1.26). Regarding the distribution of MHRIs count, 484 participants (27.47%) had no MHRIs, 560 (31.78%) had one health-risk indicator, and 718 (40.75%) had two or more MHRIs. Alcohol use (42.17%), insomnia symptoms (28.38%), and frequent picky eating (21.74%) were the most prevalent indicators, whereas Internet gaming disorder (2.89%) and smoking (7.38%) were relatively less common. The prevalence of individual health-risk indicators across the three waves is presented in Supplementary Table S1.

### Descriptive statistics, correlations, and longitudinal measurement invariance

Descriptive statistics and the correlation matrix are presented in Table 2. Psychological distress scores were positively correlated across the three waves, with correlations ranging from *r* = 0.454 to 0.629 (all *p* < 0.001). MHRIs count was also positively correlated across the three waves, with correlations ranging from *r* = 0.330 to 0.523 (all *p* < 0.001). MHRIs count and psychological distress scores at different waves were positively correlated, with correlations ranging from *r* = 0.237 to 0.372 (all *p* < 0.001).

**Table 2:** Descriptive statistics and correlation matrix for MHRIs count and psychological distress.

No.	Variable	Mean	SD	1	2	3	4	5	6
1	T1 MHRIs count	1.41	1.26	—					
2	T2 MHRIs count	1.55	1.32	0.523^***^	—				
3	T3 MHRIs count	1.61	1.37	0.330^***^	0.512^***^	—			
4	T1 psychological distress	28.46	16.54	0.282^***^	0.301^***^	0.253^***^	—		
5	T2 psychological distress	30.35	18.25	0.299^***^	0.372^***^	0.312^***^	0.629^***^	—	
6	T3 psychological distress	31.49	19.12	0.237^***^	0.328^***^	0.344^***^	0.454^***^	0.605^***^	—

MHRIs count, number of co-occurring multiple health-risk indicators; SD, standard deviation. Psychological distress was assessed using the DASS-21 total score. ^***^p < 0.001.

The configural, metric, and threshold invariance models for the DASS-21 all showed good fit. In comparisons of adjacent models, ΔCFI and ΔRMSEA did not exceed 0.002, satisfying the prespecified criteria and supporting threshold invariance across the three waves. These findings support longitudinal comparisons of psychological distress scores (Supplementary Table S2). University-level ICCs ranged from 0.003 to 0.012, whereas total within-class ICCs ranged from 0.026 to 0.042 (Supplementary Table S3), indicating relatively limited clustering at the university and class levels.

### Longitudinal trajectories of MHRIs count and psychological distress

Separate unconditional LGMs for MHRIs count and psychological distress both showed good fit (Table 3).

**Table 3:** Model fit indices for the unconditional latent growth models.

Model	χ^2^	df	*P*	CFI	TLI	RMSEA	SRMR
MHRIs count	3.345	1	0.067	0.998	0.993	0.036	0.010
Psychological distress	1.556	1	0.212	0.999	0.998	0.018	0.006

For MHRIs count, the intercept mean was 1.416 and the mean of the slope factor was 0.103 (*p* < 0.001), indicating a modest increase over time. The intercept and slope variances were 1.165 and 0.328, respectively, and both were statistically significant (both *p* < 0.001), indicating individual differences in initial levels and rates of change. The intercept and slope factors were negatively correlated (*r* = −0.484, *p* < 0.001).

For psychological distress, the intercept mean was 28.500 and the mean of the slope factor was 1.546 (*p* < 0.001), indicating an increase over time. The intercept and slope variances were 236.080 and 56.846, respectively, and both were statistically significant (both *p* < 0.001), indicating individual differences in initial levels and rates of change. The intercept and slope factors were negatively correlated (*r* = −0.399, *p* < 0.001). Detailed parameter estimates and model paths are presented in Table 4 and Figure 1A and B.

**Table 4:** Parameter estimates from the unconditional latent growth models.

Model	Parameter	Estimate (95% CI)	SE	*P*	Standardized correlation(95% CI)
MHRIs count	Intercept mean	1.416 [1.358, 1.474]	0.030	<0.001	—
	Slope mean	0.103 [0.067, 0.138]	0.018	<0.001	—
	Intercept variance	1.165 [1.006, 1.324]	0.081	<0.001	—
	Slope variance	0.328 [0.259, 0.397]	0.035	<0.001	—
	Intercept–slope covariance	−0.299 [−0.384, −0.214]	0.043	<0.001	−0.484 [−0.561, −0.408]
Psychological distress	Intercept mean	28.500 [27.724, 29.277]	0.396	<0.001	—
	Slope mean	1.546 [1.107, 1.985]	0.224	<0.001	—
	Intercept variance	236.080 [201.713, 270.448]	17.535	<0.001	—
	Slope variance	56.846 [42.552, 71.139]	7.292	<0.001	—
	Intercept–slope covariance	−46.223 [−63.239, −29.206]	8.682	<0.001	−0.399 [−0.491, −0.307]

CI, confidence interval; SE, standard error. Estimates and their 95% confidence intervals are unstandardized. Standardized correlations and corresponding 95% confidence intervals are reported only for the associations between the intercept and slope factors.

**Figure 1:**
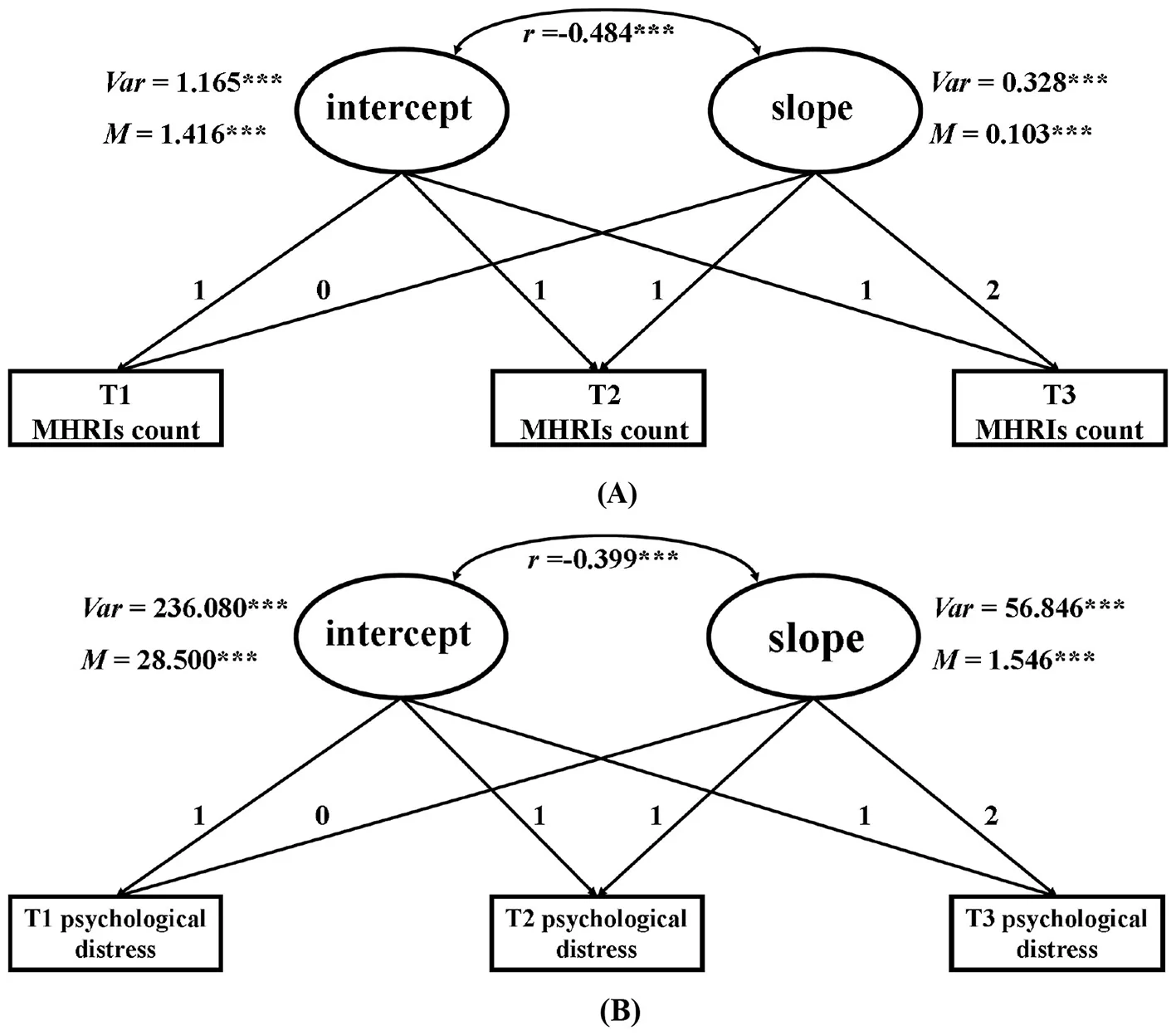
Unconditional latent growth models for **(A)** MHRIs count and **(B)** psychological distress. *M* and Var denote the latent-factor mean and variance, respectively; both are unstandardized estimates. *r* denotes the standardized correlation between the intercept and slope factors. All intercept-factor loadings were fixed at 1, and the slope-factor loadings were fixed at 0, 1, and 2 for T1, T2, and T3, respectively. ***p < 0.001.

### Cross-lagged associations between MHRIs count and psychological distress

After sex, household registration, campus residence status, only-child status, and adverse childhood experiences were included as baseline covariates, the CLPM of MHRIs count and psychological distress demonstrated acceptable-to-good fit: χ^2^(24) = 121.015, *p* < 0.001, CFI = 0.969, TLI = 0.942, RMSEA = 0.048, 90% CI [0.040, 0.057], and SRMR = 0.036. All autoregressive paths were positive. The standardized path coefficients for MHRIs count from T1 to T2 and from T2 to T3 were 0.475 and 0.459, respectively, whereas those for psychological distress were 0.592 and 0.561, respectively (all *p* < 0.001). Within-wave residual correlations between MHRIs count and psychological distress were positive at all three waves, ranging from *r* = 0.161 to 0.212 (all *p* < 0.001).

The cross-lagged paths showed that T1 psychological distress positively predicted T2 MHRIs count (β = 0.167, p <0.001), and T2 psychological distress positively predicted T3 MHRIs count (β = 0.141, *p* < 0.001). In the reverse direction, T1 MHRIs count positively predicted T2 psychological distress (β = 0.132, *p* < 0.001), and T2 MHRIs count positively predicted T3 psychological distress (β = 0.119, *p* < 0.001). The bidirectional cross-lagged paths were significant and directionally consistent across both intervals, indicating a stable positive bidirectional longitudinal association between MHRIs count and psychological distress. Detailed standardized parameter estimates are presented in Table 5, and the model paths are shown in Figure 2.

**Table 5:** Standardized parameter estimates from the cross-lagged panel model.

Path type	Path	β (95% CI)	SE	*P*
Autoregressive	T1 psychological distress → T2 psychological distress	0.592 [0.554, 0.630]	0.019	<0.001
	T2 psychological distress → T3 psychological distress	0.561 [0.520, 0.601]	0.021	<0.001
	T1 MHRIs count → T2 MHRIs count	0.475 [0.435, 0.516]	0.021	<0.001
	T2 MHRIs count → T3 MHRIs count	0.459 [0.417, 0.501]	0.021	<0.001
Within-wave residual correlation	T1 psychological distress ↔ T1 MHRIs count	0.212 [0.163, 0.261]	0.025	<0.001
	T2 psychological distress ↔ T2 MHRIs count	0.194 [0.146, 0.242]	0.025	<0.001
	T3 psychological distress ↔ T3 MHRIs count	0.161 [0.112, 0.211]	0.025	<0.001
	T1 psychological distress → T2 MHRIs count	0.167 [0.120, 0.214]	0.024	<0.001
Cross-lagged	T2 psychological distress → T3 MHRIs count	0.141 [0.095, 0.188]	0.024	<0.001
	T1 MHRIs count → T2 psychological distress	0.132 [0.091, 0.173]	0.021	<0.001
	T2 MHRIs count → T3 psychological distress	0.119 [0.075, 0.164]	0.023	<0.001

β represents the standardized parameter estimate; CI, confidence interval; SE, standard error.

**Figure 2:**
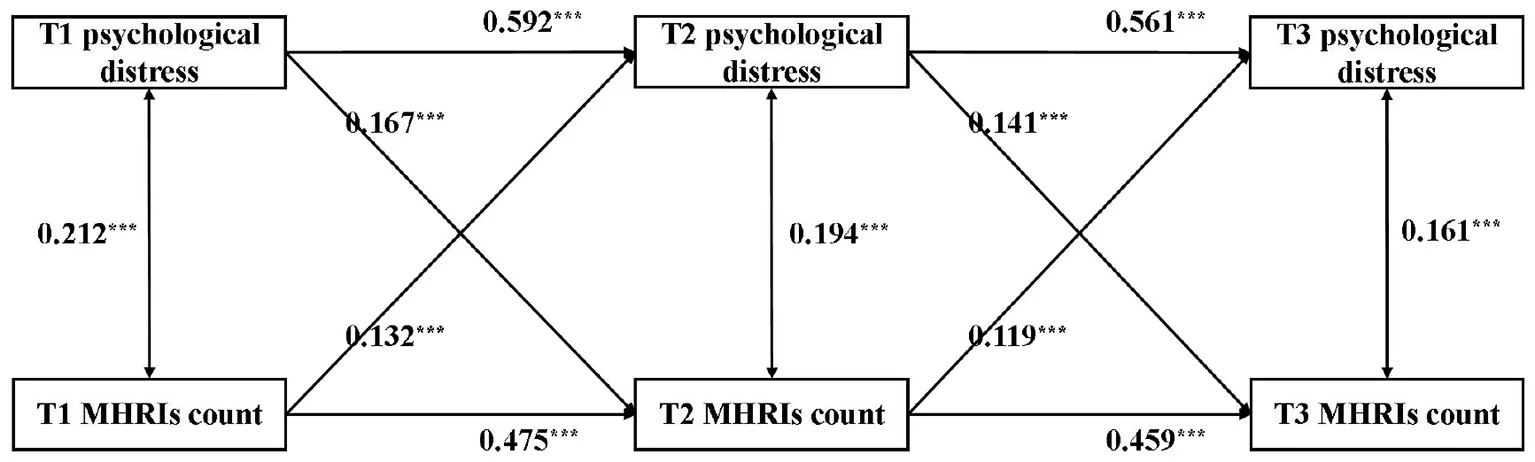
Cross-lagged panel model of MHRIs count and psychological distress. Values are standardized parameter estimates. Double-headed arrows indicate within-wave residual correlations between MHRIs count and psychological distress. T1 MHRIs count and T1 psychological distress were regressed on sex, household registration, campus residence status, only-child status, and adverse childhood experiences. ***p < 0.001.

## Discussion

Using three-wave follow-up data, this study examined developmental changes in MHRIs count and psychological distress among university students and their bidirectional longitudinal association. Both MHRIs count and psychological distress increased over the 1-year follow-up, with significant individual differences in initial levels and rates of change. Psychological distress positively predicted subsequent MHRIs count, and MHRIs count positively predicted subsequent psychological distress. These findings extend evidence previously centered on individual behaviors or cross-sectional associations to the co-occurrence of multiple health risks and a bidirectional longitudinal process, providing new evidence for understanding the dynamic links between psychological and behavioral health risks among university students.

At baseline, 40.75% of participants reported two or more MHRIs, and alcohol use (42.17%), insomnia symptoms (28.38%), and frequent picky eating (21.74%) were among the most prevalent indicators. These findings are broadly consistent with previous studies showing that multiple health-risk indicators are common among university students, although prevalence estimates vary depending on the types of indicators included and the criteria used for classification (2, 33). University students are in emerging adulthood, a transitional period between adolescence and adulthood during which identity exploration, emotion regulation, and self-control continue to develop. Some individuals may prioritize immediate experiences and short-term rewards while giving insufficient consideration to the long-term health consequences of their behavior, increasing the likelihood that different risks co-occur or accompany one another (34, 35). In addition, greater autonomy and reduced family supervision during university give students more discretion over sleep schedules, dietary choices, substance use, and digital media use, while they must also manage academic demands, interpersonal relationships, and uncertainty about future development. The combined influence of these individual and environmental factors may promote the accumulation of multiple health risks (36, 37). University health-risk management should therefore not focus solely on students who already have multiple health risks but should also use continuous monitoring to identify those whose risk profiles are worsening over time.

Psychological distress also increased across the three measurement occasions, suggesting that distress may accumulate as students progress through university. This finding differed from a previous longitudinal study in which depression, anxiety, and stress levels remained generally stable among university students (38). The discrepancy may reflect differences in regional culture, social context, and the timing of data collection. Unlike previous samples, participants in this study were enrolled at universities in three Chinese provinces, and data were collected in 2025–2026. As university life progressed, students may have faced sustained pressures related to academic demands, interpersonal adjustment, career decisions, and employment uncertainty, potentially contributing to increasing psychological distress (39). Differences in sample composition, year-of-study distribution, and follow-up intervals may also influence mean changes in psychological distress (40). University mental health services should therefore extend beyond screening at enrollment or at a single time point and include periodic assessment and continuing follow-up to identify students whose psychological distress is increasing.

The cross-lagged analysis showed that a higher MHRIs count positively predicted subsequent psychological distress, broadly consistent with previous evidence (10). First, lifestyle patterns characterized by substance use or unhealthy eating have been associated with subsequent increases in depression, anxiety, and stress symptoms (41). Second, experiences and behaviors such as bullying victimization and NSSI often accompany impaired interpersonal relationships, reduced social support, and negative self-evaluation, which may increase the risk of psychological distress (42, 43). In addition, problematic internet use may exacerbate depression, anxiety, and stress symptoms by disrupting sleep rhythms, displacing in-person social interaction, and impairing daily functioning (44). When multiple health-risk indicators and related risks co-occur, their adverse consequences for physical, psychological, and social functioning may have additive or compounding effects, thereby contributing to greater subsequent psychological distress.

Psychological distress also positively predicted subsequent MHRIs count, suggesting that distress may not only accompany multiple behavioral risks but may also be involved in their further accumulation. Persistent depression, anxiety, and stress symptoms may deplete cognitive and emotion-regulation resources and weaken the ability to maintain healthy routines, inhibit impulses, and make health decisions with long-term consequences in mind (45). University students experiencing psychological distress may also rely more heavily on alcohol use, gaming, social media use, or other strategies that offer immediate relief. These strategies may not resolve ongoing stress and could gradually expand into the co-occurrence of multiple health risks (46, 47). The directionally consistent bidirectional predictive associations across both intervals suggest a potentially mutually reinforcing dynamic process between MHRIs count and psychological distress. University health promotion should therefore integrate psychological distress screening with assessment of multiple health-risk indicators rather than treating psychological and behavioral risks separately. From a public health perspective, university-based interventions may benefit from shifting from single-risk prevention toward integrated risk management approaches. Early identification of students with accumulating risk profiles, combined with targeted support for sleep problems, substance use, digital media-related problems, and psychological distress, may help prevent the further accumulation of health risks. Coordinated strategies involving screening, health education, behavioral counseling, and accessible psychological services may provide a more comprehensive approach to promoting student health.

## Strengths and limitations

This study has several strengths. First, separate LGMs characterized the overall trajectories of MHRIs count and psychological distress and identified individual differences in their initial levels and rates of change, supporting a developmental perspective on psychological and behavioral health risks among university students. Second, the CLPM enabled examination of temporal ordering and bidirectional longitudinal associations between MHRIs count and psychological distress, providing longitudinal evidence for a potentially mutually reinforcing process. Finally, by considering multiple behavioral risks together with psychological distress, this study provides evidence that may inform integrated risk screening, targeted prevention, and efforts to improve the health and quality of life of university students.

Several limitations should be acknowledged. First, although the three-wave longitudinal design allowed examination of developmental trajectories and temporal associations, the relatively short follow-up period limited assessment of long-term changes and potential nonlinear patterns. Future studies with longer follow-up and additional measurement occasions are warranted. Second, self-reported questionnaires may have introduced recall and reporting biases; future research incorporating objective behavioral measures or multiple informants is encouraged. Third, participants were recruited from higher education institutions in three Chinese provinces, which may limit generalizability to other populations and settings.

## Data Availability

The raw data supporting the conclusions of this article will be made available by the authors, without undue reservation.
