# Supplementary material for: Longitudinal associations between multiple health-risk indicators and psychological distress among university students: a three-wave cross-lagged study
Source: Front Public Health. 2026 Sep 23;14:1958845. doi: 10.3389/fpubh.2026.1958845 (PMC13642685; doi:10.3389/fpubh.2026.1958845)
Supplement: Supplementary file 1 [file Data_Sheet_1.pdf]

Supplementary Material

Supplementary Table S1. Prevalence of individual multiple health-risk indicators across the three waves and distribution of MHRIs count at baseline

Panel A. Prevalence of individual MHRIs across the three waves

Indicator	T1, n (%)	T2, n (%)	T3, n (%)
Smoking	130 (7.38)	147 (8.34)	157 (8.91)
Alcohol use	743 (42.17)	789 (44.78)	819 (46.48)
Frequent picky eating	383 (21.74)	425 (24.12)	440 (24.97)
Insomnia symptoms	500 (28.38)	551 (31.27)	573 (32.52)
Traditional bullying victimization	198 (11.24)	203 (11.52)	194 (11.01)
Cyberbullying victimization	134 (7.60)	160 (9.08)	162 (9.19)
Nonsuicidal self-injury	223 (12.66)	249 (14.13)	264 (14.98)
Internet gaming disorder	51 (2.89)	61 (3.46)	67 (3.80)
Problematic social media use	118 (6.70)	152 (8.63)	159 (9.02)

Note: Values are presented as n (%). The analytic sample comprised 1,762 university students at each wave. T1, baseline; T2, 6-month follow-up; T3, 12-month follow-up.

Panel B. Distribution of MHRIs count at baseline

Baseline number of MHRIs	n	%
0	484	27.47
1	560	31.78
2	394	22.36
3	203	11.52
4	90	5.11
5	25	1.42
6	4	0.23
7	2	0.11
Total	1,762	100

Note: MHRIs, multiple health-risk indicators. Co-occurring MHRIs were defined as the presence of two or more MHRIs.

Supplementary Table S2. Longitudinal measurement invariance of the three-factor DASS-21 across the three measurement waves

Model	χ^2	df	p	CFI	TLI	RMSEA	SRMR	ΔCFI	ΔRMSEA
Configural invariance	2316.087	1854	<0.001	0.994	0.994	0.012	0.024	—	—
Metric invariance	2262.156	1890	<0.001	0.995	0.995	0.011	0.026	0.001	0.001
Threshold invariance	2589.970	2010	<0.001	0.993	0.993	0.013	0.026	0.002	0.002

Note: The DASS-21 was specified as a correlated three-factor model comprising depression, anxiety, and stress at each wave. Models were estimated using the weighted least squares mean and variance adjusted estimator. Changes in CFI and RMSEA were calculated relative to the immediately preceding model. Longitudinal measurement invariance was considered supported when ΔCFI < 0.010 and ΔRMSEA < 0.015. CFI, comparative fit index; TLI, Tucker–Lewis index; RMSEA, root mean square error of approximation; SRMR, standardized root mean square residual; DASS-21, Depression Anxiety Stress Scales-21.

Supplementary Table S3. Intraclass correlation coefficients for MHRIs count and psychological distress across the three measurement waves

Variable	University-level ICC	Class-within-university component	Total within-class ICC
MHRIs count T1	0.003	0.023	0.026
MHRIs count T2	0.004	0.025	0.029
MHRIs count T3	0.004	0.022	0.026
Psychological distress T1	0.008	0.027	0.035
Psychological distress T2	0.012	0.030	0.042
Psychological distress T3	0.006	0.029	0.036

Note: ICC, intraclass correlation coefficient. The class-within-university component represents the additional variance attributable to classes after accounting for university-level clustering. Total within-class ICC represents the combined university- and class-level clustering for students within the same class. T1, baseline; T2, 6-month follow-up; T3, 12-month follow-up.
